# Moderating Multiple Mediation Model of the Impact of Inclusive Leadership on Employee Innovative Behavior

**DOI:** 10.3389/fpsyg.2021.666477

**Published:** 2021-08-11

**Authors:** Hui Wang, Min Chen, Xiangqing Li

**Affiliations:** Human Resource Management Department, Business School, Xiangtan University, Xiangtan, China

**Keywords:** inclusive leadership, psychological safety, creative self-efficacy, innovation rewards, employee innovative behavior, moderating multiple mediation model

## Abstract

Leadership is an important antecedent variable of employee innovative behavior. In previous studies, the influence of inclusive leadership on employee innovative behavior mainly focus on one mediating variable, which may lead to a deviation in parameter estimation due to the omission of other mediating variables. According to the social cognitive theory and motivation theory and from the perspective of cognitive–motivation integration, this study establishes a moderating multi-mediation model to understand the impact of inclusive leadership on employee innovative behavior. Psychological safety and creative self-efficacy are used as mediating variables, and innovation rewards are used as moderating variables. The data are collected from 418 employees of the manufacturing industry in China. The results show that, at first, inclusive leadership has a positive effect on employee innovative behavior. Secondly, both psychological safety and creative self-efficacy play partial mediating effects between inclusive leadership and employee innovative behavior, with the effect of the former being significantly smaller than that of the latter. Thirdly, innovation rewards positively moderate the relationships of “psychological safety—employee innovative behavior” and of “creative self-efficacy—employee innovative behavior.” Fourthly, innovation rewards positively moderate the indirect effect of inclusive leadership on employee innovative behavior through psychological safety and creative self-efficacy. These findings are not only helpful to expand how inclusive leadership influences the innovative behavior of employees but also provides some suggestions for enterprise innovation development.

## Introduction

In a competitive environment characterized by economic globalization, rapid technological change and shortened product renewal, organizational innovation has been regarded as the crucial facilitator for enterprise to gain competitive advantages and realize the sustainable development. Thus, seeking effective measures to stimulate employee innovative behavior is not only the main goal pursued by enterprises but also an important research topic. As a result, studying the drivers of employee innovative behavior is vital (Scott and Bruce, 1994).

Employee innovative behavior is a complete process in which individuals generate, promote, and apply new ideas at work (Scott and Bruce, 1994). In recent decades, antecedents of employee innovative behavior, including leadership, work climate, job characteristics and demand, individual differences, and personality, have been examined (Anderson and West, 1998; Janssen, 2000; Baer and Frese, 2002; Anderson et al., 2004; Zhou and Hoever, 2014; Zlatanović and Mulej, 2015; Franco and Haase, 2017; Vandavasi et al., 2019). Among several possible influential factors, leadership has been identified as an influential driver of employee innovative behavior (Zhou and Hoever, 2014). Such as, authentic leadership (e.g., Laguna et al., 2019), transformational leadership (e.g., Bednall et al., 2018), and servant leadership (e.g., Zhu and Zhang, 2019; Iqbal et al., 2020) have been proven to affect employee innovative behavior. As Fletcher (2004) and Uhl-Bien (2006) called for further research on relational leadership, changing economic conditions require leaders to be more attentive to relationship building to create a more motivated workforce (Uhl-Bien, 2006). In addition, some researchers have garnered their interest in one kind of relational leadership, inclusive leadership, which differs from transformational leadership, servant leadership, and authentic leadership by its explicit focus on the openness, availability, and accessibility of leaders to meet the needs of employees (Bandura, 1977). Inclusive leaders accept and respect employee differences, recognize employee effort and value, and encourage employee participation (Edmondson and Lei, 2014). Inclusive leadership helps employees to participate in important processes with ease and confidence and paves a path for them to promote and apply their creative ideas (Choi et al., 2015). Therefore, inclusive leadership can be asserted as a primary factor behind the promotion of employee innovative behavior (Carmeli et al., 2010; Javed et al., 2017, 2018; Fang et al., 2019; Wang et al., 2019). Moreover, under the background of economic globalization, the increase of cross-regional population flow leads to constant improvement in the degree of employee diversity (MorBarak, 2014; Benson et al., 2016). This notion requires inclusive leadership, which assures that all team members feel they are treated respectfully and fairly, are valued and sense that they belong, and are confident and inspired. However, research on the effects of inclusive leadership on employee innovative behavior has been scarce, especially on the mediating mechanism of the influence of inclusive leadership on employee innovation behavior.

Extant studies have examined the relationship between inclusive leadership and employee innovative behavior using various mediating mechanisms such as psychological safety (Carmeli et al., 2010; Javed et al., 2017; Zhu et al., 2019; Mansoor et al., 2020), leader–member exchange (Javed et al., 2018), psychological empowerment (Randel et al., 2017; Javed et al., 2018), organizational commitment (Choi et al., 2015), psychological capital (Preacher and Hayes, 2008; Fang et al., 2019), and perceived organizational support (Qi et al., 2019). Given that employee innovative behavior is a non-routine method that typically avoids traditional methods in approaching work, explores, and implements new work means, then employees need psychological safety to advance innovation processes (Edmondson and Lei, 2014). Therefore, the relationship between inclusive leadership and employee innovative behavior is mainly examined to be mediated by psychological safety (Carmeli et al., 2010; Javed et al., 2017; Zhu et al., 2019; Mansoor et al., 2020). These studies rely on the leader–member exchange theory. In addition, Bandura (1977, 2001) argued that creative self-efficacy is an important mechanism resulting in desirable outcomes such as employee innovative behavior. More recently, Javed et al. (2020) investigated the role of creative self-efficacy as a mediating mechanism in the relationship between inclusive leadership and employee innovative behavior based on the social cognitive theory.

By reviewing previous literature studies, we find that most studies only use one mediating variable to explain the relationship between inclusive leadership and employee innovative behavior. However, the model of one mediating variable is prone to produce a deviation in parameter estimation due to the omission of other mediating variables. Furthermore, employee innovative behavior is full of risks and challenges. An individual who exhibits innovative behavior, he/she considers both “Do I have the ability to do this?” and “Is it safe to do?” As stated in Edmondson and Lei (2014), psychological safety is a state where individuals feel that it is safe to take interpersonal risks in workplace. In the present study, psychological safety is considered as an individual cognition, which reflects the belief that showing risky behaviors do not cause personal harm or interpersonal threats on self-image, career, or status of individuals, whereas creative self-efficacy refers to the confidence in individual ability to creatively resolve problems and achieve innovative results (Tierney and Farmer, 2002). Both psychological safety and creative self-efficacy belong to the category of “cognition.” Thus, based on the social cognitive theory, inclusive leadership affects employee innovative behavior through the employee cognition, that is, both psychological safety and creative self-efficacy mediate the relationship between inclusive leadership and employee innovative behavior. In this study, a parallel dual mediation model is constructed to explore the effect of inclusive leadership on employee innovative behavior and to further compare the mediating effects of psychological safety and creative self-efficacy.

In addition, innovation is not only a cognitive activity but also a behavior driven by motivation (Jong and Hartog, 2007). Given that the innate interest of an individual is limited, most human behaviors must be cultivated, including motivation. For those behaviors who lack internal motivation, the use of external stimulation is important to stimulate motivation. In management practice, many enterprises encourage employee innovative behavior by formulating salary incentives such as employee stock ownership plans. However, limited research has integrated the two different theoretical perspectives of cognition and motivation to investigate employee innovative behavior. Innovation rewards, which are defined as “the rewards given by organization, which are based on the innovative performance of employees,” can enhance the motivation of employees to perform innovative behavior (Malik et al., 2015; Amabile and Pratt, 2016). Therefore, based on the motivation theory, the current study considers innovation rewards as a moderator to explore the boundary conditions of inclusive leadership in affecting employee innovative behavior.

**Figure 1:**
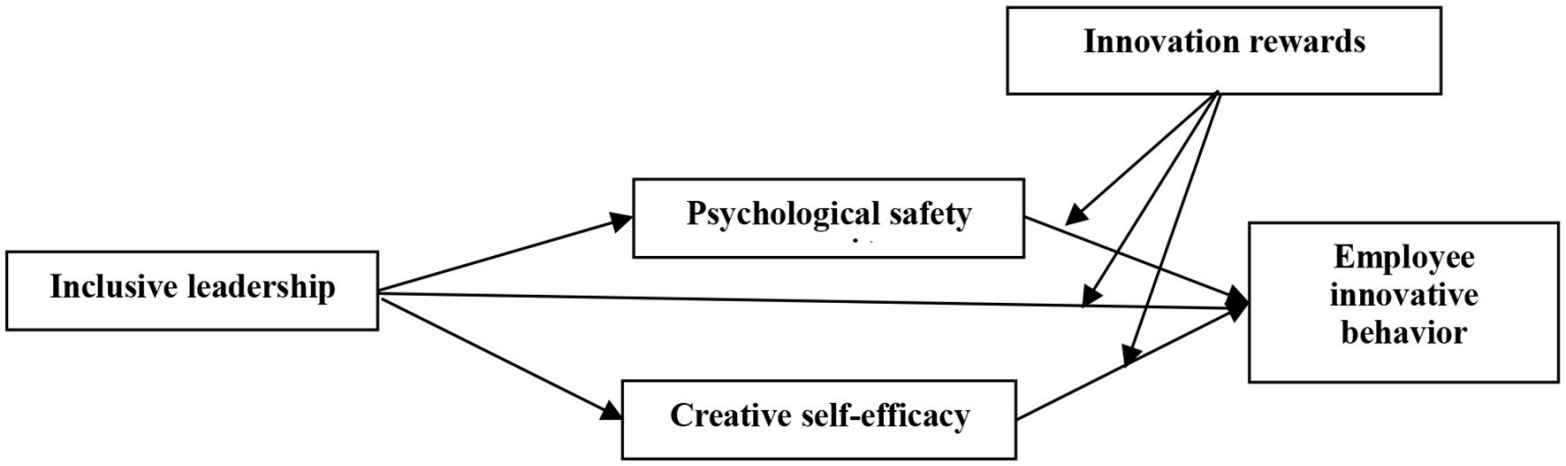
Theoretical model.

All in all, this study integrates the social cognition theory and motivation theory to construct a moderated multiple mediation model of inclusive leadership affecting employee innovative behavior, which uses psychological safety and creative self-efficacy as mediating variables and innovation rewards as a moderating variable. The theoretical framework is presented in Figure 1. Thus, this study has the following contributions. Firstly, in previous studies, the mediating role of psychological safety is examined based on the leader–member exchange theory, whereas this study uses the social cognitive theory as a basis. Secondly, this study constructs a dual mediation model that psychological safety and creative self-efficacy are selected as mediating variables to explain how inclusive leadership affects employee innovative behavior. Furthermore, a comparative analysis of two mediating effects is used to distinguish the differences between these two mediator variables. Furthermore, a comparative analysis of two mediating effects is used to distinguish the differences between these two mediator variables. Thirdly, this study explores the moderating role of innovation rewards in the relationship of inclusive leadership and employee innovative behavior based on the motivation theory. Furthermore, social cognition and motivation theories are integrated to understand the boundary effect of the mediation mechanism. This integration not only makes up for the research deficiency on the influence of cognition–motivation interaction on employee innovative behavior but also broadens the literature on the boundary effect between inclusive leadership and employee innovative behavior. Finally, this study is conducted in China, which has notable differences from western contexts. The Chinese culture advocates spiritual qualities such as “harmony without uniformity and inclusiveness” and “tolerance is a virtue,” which contains the idea of “inclusiveness.” Some researchers have focused on inclusive leadership in the Chinese context (Fang et al., 2019; Qi et al., 2019; Wang et al., 2019; Ye et al., 2019; Zhu et al., 2019). This study extends the literature on the relationship of inclusive leadership and employee innovative behavior in the Chinese context.

## Theoretical Background and Hypotheses

### Inclusive Leadership and Employee Innovative Behavior

Employee innovative behavior has been defined from different perspectives, but most studies on innovative behavior defined innovative behavior from the perspective of an innovation process. From this perspective, innovative behavior is usually understood as a process consisting of idea generation, idea promotion, and idea realization (Scott and Bruce, 1994; Janssen, 2000; Kleysen and Street, 2001). In the first stage, individuals recognize problems and identify novel ideas and solutions. In the second stage, employees seek sponsorship and build coalitions to support the new ideas. In the third stage, the employees complete the process when they gain enough support to produce a prototype that can be diffused and institutionalized (Scott and Bruce, 1994).

Among several possible influential factors, leadership has been identified as an influential driver of employee innovative behavior (Zhou and Hoever, 2014). Nembhard and Edmondson (2006) originally proposed the concept of inclusive leadership in the field of management, and defined this as the “words and deeds by a leader or leaders that indicate an invitation and appreciation for the contribution of others.” Subsequently, Hollander (2014) defined inclusive leadership as a win–win situation with a common goal and vision of interdependent relationships. Saz-Carranza and Ospina (2011) described that an inclusive leader is valuable, and is someone who accepts staff at all levels in the organization and is responsible for results. Furthermore, in the context of Chinese culture, inclusive leadership not only accepts and respects employee differences, recognizes employee effort and value, and encourages employee participation, but also accommodates employee error (Zheng et al., 2018). Compared with the other conceptually related forms of leadership, inclusive leadership holds a unique nature of acceptance, belongingness, uniqueness, and inclusiveness (Randel et al., 2017). Inclusive leadership contains three dimensions: (a) leaders tolerated the views and failures of employees through attentive listening, rationally tolerate their errors, and provided encouragement and guidance to support staff when the latter makes mistakes; (b) leaders recognize and train employees by respecting and focusing on employee training and praising achievements; and (c) leaders treat employees fairly, consider their needs and interests, show a fair attitude toward employees, and ensure that they share earnings.

Inclusive leadership enhances employee innovative behavior in several ways. Firstly, inclusive leaders accept and respect employee differences not only in their professional skills, values, and religious beliefs but also in their diverse ideas (Zheng et al., 2018). Innovation means breaking the rules. “Diversity tolerance,” the characteristic of inclusive leadership, enables employees to bring their differentiated knowledge and information into full play. Such a collision of heterogeneous knowledge in the team is conducive to the emergence of new ideas and stimulates employee innovative behavior. Secondly, inclusive willingness of leaders to listen to employees and providing them with an open and trustworthy “communication climate” improve employee identification to the organization (Elsaied, 2020), which, in turn, enhances their organizational commitment and stimulates employee innovative behavior (Choi et al., 2015). Thirdly, inclusive leaders involve followers in decision-making and ensure their availability to help employees in every step; therefore, employees gain a chance to enhance their creative thinking (Sharifirad and Ataei, 2012), and supporting resources for innovation (Javed et al., 2018; Bannay et al., 2020). This capability of the generation of new thoughts is considered as a progression toward innovative behavior (Min, 2004). In addition, inclusive leaders recognize employee contributions and pay attention to cultivating employee development (Dwertmann and Boehm, 2016). According to the principle of reciprocity, the leader support perceived by employees, in turn, stimulates them to exhibit more innovative behavior to reward their organization (Qi et al., 2019). Furthermore, inclusive leaders are good at listening to the opinions of employees, which make employees feel being respected. The stronger the employees' feeling of being respected by their leaders, the higher the employees' psychology empowerment (Wang et al., 2019), and the more innovative behavior would be made by employees. Finally, inclusive leaders embrace employee mistakes at work. “Fault tolerance” sends a hint of error resilience to employees, which makes them feel safe to innovate, remove the work ethic that reduces the rate of mistakes, and dare to come up with new ideas and put them into action (Fuller et al., 2006). Based on the above discussion, the following hypothesis is proposed.

***Hypothesis 1:****Inclusive leadership is positively related to employee innovative behavior*.

### Psychological Safety as a Mediator Between Inclusive Leadership and Employee Innovative Behavior

Kahn (1990) defined psychological safety as “the subjective perception that individuals feel that they have ability to show themselves without fear of negative consequences for self-image, career, or status.” Recently, psychological safety was conceptualized as a state where individuals feel that taking interpersonal risks in the workplace is safe (Edmondson and Lei, 2014). Therefore, in the present study, psychological safety is considered as an individual cognition that reflects beliefs that showing risky behaviors do not cause personal harm or interpersonal threats on the self-image, career, or status of individuals (Kahn, 1990; Edmondson and Lei, 2014).

According to the social cognitive theory, employee behavior is influenced by the individual's cognitive and organizational environment factors. Given that the leadership style is an important antecedent variable of psychological safety (Tynan, 2005), then the openness and inclusiveness of the management can promote the employee perception of psychological safety. Due to the uncertainty and complexity in innovation and its risks, psychological safety can provide employees with “have the ability to do” motivation. Based on this notion, this study concludes that when leaders show inclusive traits and behaviors in innovation, employees improve their perceptions of organizational environment security and exhibit more innovative behaviors.

On one hand, inclusive leadership can effectively enhance the psychological safety of employees, which, in an organization, depends to a great extent on the respect and trust of others in the work environment, especially the supervisors who evaluate employee performance (Kaiser et al., 2008). Previous literature studies have confirmed that inclusive leadership has a positive effect on psychological safety (Nembhard and Edmondson, 2006; Carmeli et al., 2010; Javed et al., 2017; Ye et al., 2019; Zhu et al., 2019). Firstly, inclusive leaders attempt to encourage employees to participate in decision-making, give employees the freedom to decide their work activities on themselves, and ensure that employees are not punished for engaging in challenging tasks. As a consequence, employees will experience greater psychological safety (Nembhard and Edmondson, 2006; Carmeli et al., 2010). Secondly, inclusive leaders displaying a high level of availability and accessibility to employees send a clear signal that undertaking risky behaviors is safety without concerning the negative consequences, which enhances the psychological safety of employees (Carmeli et al., 2010; Javed et al., 2017). Thirdly, inclusive leaders, such as a positive interaction with employees and build a closer connection with employees, actively reduce the psychological distance with employees and effectively improve the psychological safety of employees(Ye et al., 2019; Zhu et al., 2019). Furthermore, in the Chinese context, inclusive leadership has the connotation of “fault tolerance,” which means that when an employee makes a mistake, the leader forgives and does not completely deny the employee because of one mistake. This “fault-tolerant” behavior leads employees to experience greater psychological safety.

On the other hand, psychological safety can stimulate employees to exhibit innovative behavior. First of all, innovation means breaking the mold, which is a highly uncertain activity. When innovating, employees face the risk of failure (Tierney and Farmer, 2002), which makes them cautious about such behavior. Psychological safety is an important indicator of the perceived risks of an individual, which affects the individual's “can do” motivational state that prompts innovative behavior (Parker et al., 2010). When employees have a higher sense of psychological safety, the “can do” motivation of employees becomes stronger, then they become more innovative behaviors (Mueller and Kamdar, 2011). In other words, employees who perceive higher psychological safety worry less about the risk of innovation and create more innovation at work (Chen et al., 2020). In addition, employees with high psychological safety trust their colleagues and leaders and do not have to worry about leaders' criticisms of new ideas. Therefore, they share or collect knowledge to and from colleagues (Kessel et al., 2012). This kind of knowledge sharing can break through the limitations of employee's inherent thinking, enable them to generate more new ideas and prompting innovative behavior.

In conclusion, based on the social cognitive theory, this study follows the effect path of “environmental perception–psychological cognition–behavior,” and argues that inclusive leadership prompts employee innovative behavior by enhancing the psychological safety of employees. Thus, the second hypothesis is proposed.

***Hypothesis 2****: Psychological safety plays a mediating role between inclusive leadership and employee innovative behavior*.

### Creative Self-Efficacy as a Mediator Between Inclusive Leadership and Employee Innovative Behavior

Self-efficacy is defined as “the confidence that an individual has the ability to execute the actions needed to produce certain achievements” (Bandura et al., 1997). According to the social cognitive theory (Hollander, 2014), self-efficacy plays a crucial role between the external environment and an individual's behavior. Creative self-efficacy is a specific form of self-efficacy that refers to an individual's confidence in the ability to creatively resolve problems and achieve innovative results (Tierney and Farmer, 2002). As such, this study argues that inclusive leadership improves the creative self-efficacy of employees, thus enhancing their willingness to exhibit innovative behavior.

On one hand, inclusive leadership can enhance the creative self-efficacy of employees. Self-efficacy is affected by four factors: work attainment, vicarious experience, verbal persuasion, and psychological state (Bandura, 1982). Firstly, inclusive leadership encourages employees to participate, listens to their opinions, and demonstrates trust in their competence. The trust of superiors in turn enhances the work attainment of employees. Therefore, self-experience enhances the creative self-efficacy of employees. Secondly, vicarious experience usually comes from the work performance of an influential superior (Yang and Cheng, 2009). Inclusive leaders provide guidance and assistance when employees encounter difficulties. By observing the behaviors of leaders, employees can learn relevant knowledge and expertise and improve their work capacity, then improve their creative self-efficacy (Ye et al., 2018). Thirdly, inclusive leaders trust and encourage employees. Such a verbal persuasion from leaders increases the employee's confidence in completing tasks. At the same time, inclusive leaders are interested in communicating organizational goals with employees, which not only helps the latter to understand the link with their work and organizational targets but also prompts them to think that the work is meaningful, thereby enhancing their creative self-efficacy. Moreover, inclusive leaders treat employees fairly such that a harmonious atmosphere in the workplace is established, which helps employees to maintain a positive and an optimistic psychological state, then improve their creative self-efficacy (Abbas and Raja, 2015). In addition, “fault tolerance” is an important characteristic of inclusive leadership. Therefore, inclusive leaders can forgive failure or fault, which reduces the anxiety of employees about mistakes and enhances their creative self-efficacy.

On the other hand, creative self-efficacy can effectively stir up employees to exert innovative behavior. Innovation is a challenging activity that depends not only on the willingness to do it but also on the ability to do it. Therefore, as a cognitive construct of self-competence, creative self-efficacy provides employees with the motivation to “have the ability to do,” which is an important influencing factor of innovation. The important role of creative self-efficacy in the innovation behavior of individuals has been confirmed (Mielniczuk and Laguna, 2018; Newman et al., 2018; Su et al., 2019; Teng et al., 2019; Javed et al., 2020). Firstly, employees with higher creative self-efficacy have more confidence in their capabilities and are then likely to demonstrate innovative behavior by attempting more creative tasks and putting greater efforts into achieving their goals (Su et al., 2019; Yoon and Yoon, 2019). Secondly, when obstacles and difficulties arise during innovation, employees with high self-efficacy tend to adopt problem-focused coping strategies and form a behavioral guidance to adapt to changes, then taking innovative behavior to actively respond to problems. Conversely, those employees with low creative self-efficacy tend to adopt emotion-focused coping strategies and form the behavioral orientation of avoiding risks, then maintain the status quo.

In conclusion, following the effect path of “environmental perception—psychological cognitive behavior,” this study argues that when leaders exhibit “inclusive” behavior, the creative self-efficacy of employeesis enhanced. Therefore, employees dare to undertake challenging innovative activities. Therefore, the third hypothesis is proposed.

***Hypothesis 3:****Creative self-efficacy mediates the relationship between inclusive leadership and employee innovative behavior*.

Based on Hypotheses 2 and 3, combined with the social cognitive theory, both creative self-efficacy (Can I do it?) and psychological safety (How risky is it?) belong to “can do” cognition, and these two variables can mediate the relationship between inclusive leadership and employee innovative behavior. However, the extension of the two mediating effects may differ. Therefore, this study proposes a parallel dual mediation model and use a comparative analysis in the empirical analysis to distinguish the differences of these two mediating effects.

### Moderating Role of Innovation Rewards in the Relationship of Inclusive Leadership and Employee Innovative Behavior

Employee innovation behavior is the foundation of enterprise innovation and the power source of sustainable organizational development. For this reason, more and more enterprises implement innovation reward policies to stimulate employee innovative behavior. Innovation rewards refers to external rewards such as bonus, praise, recognition, or promotion given to employees for their innovation performance based on performance indicators (Byron and Khazanchi, 2012). According to the motivation theory, innovation rewards can inspire employee motivation to exhibit more innovative behaviors (Byron and Khazanchi, 2012; Malik et al., 2015). On one hand, based on an innovation reward policy, employees who implement innovative behaviors are rewarded by the organization. This positive feedback releases a signal that the organization encourages innovation and can stimulate intrinsic motivation on employees for innovation (Uco and Wiersma, 1992; Malik et al., 2015). On the other hand, innovation rewards are not only the compensation for the value of high-risk activities such as innovation but also the compensation for the efforts of employees in their work (Burris, 2012; Bhatnagar Jyotsna, 2014; Kanama and Nishikawa, 2017). This policy results in positive reinforcement, which strengthens the external motivation for employee innovation and provides employees with the motivation of “have reasons to do” through the integration of internal motivation and external motivation, then stimulates employee innovation behavior.

As discussed above, both inclusive leadership and innovation rewards are positively related to employee behavior. This study infers that innovation rewards positively moderate the relationship of inclusive leadership and employee innovative behavior. If the organization implements high innovation rewards, there is a strong positive relationship between inclusive leadership and employee innovative behavior. By contrast, under low levels of innovation rewards, even though the leader is inclusive, but employees may consider that the innovation is not encouraged by organization. Therefore, the lack of motivation prevents them from making attempts to innovate. Thus, the fourth hypothesis is proposed.

***Hypothesis 4:****Innovation rewards play a moderating role between inclusive leadership and employee innovative behavior. Compared with the low innovation rewards, the positive relationship between inclusive leadership and employee innovative behavior is stronger under high innovation rewards*.

### Moderating Role of Innovation Rewards in the Relationship of Psychological Safety and Employee Innovative Behavior

Psychological safety gives employees the recognition of “can do” while innovation rewards stimulate the employee motivation of “have reason to do.” That is to say, employees with higher psychological safety perceive the safety of engaging in risky activities such as innovation. At the same time, if the organization implements high innovation rewards, then employees are more likely to exhibit innovative behaviors that meet the organizational expectations and bring value to themselves. By contrast, under low levels of innovation rewards, employees do not gain sufficient rewards for their innovative behavior, and thus have weak willingness to innovate. Even though employees have higher psychological safety and believe that innovation in an organization is safe, they may consider that the innovation is not encouraged by organization. Therefore, the lack of motivation prevents them from making attempts to innovate. Thus, the fifth hypothesis is proposed.

***Hypothesis 5:****Innovation rewards play a moderating role between psychological safety and employee innovative behavior. Compared with the low innovation rewards, the positive relationship between psychological safety and employee innovative behavior is stronger under high innovation rewards*.

### Moderating Role of Innovation Rewards in the Relationship of Creative Self-Efficacy and Employee Innovative Behavior

Creative self-efficacy also provides employees the recognition of “can do,” whereas innovation rewards stimulate the motivation of employees of “have reason to do.” These two factors interact with each other and together influence employee innovation behavior. Innovation rewards that continuously give employees positive feedback and positive reinforcement cannot only stimulate the intrinsic motivation of employees for fulfilling their competency needs but also inspire their external motivation by giving them good expectations for the consequences (e.g., monetary rewards, praise, and post promotion) of innovation (Kris and Shalini, 2012). Under the joint effect of intrinsic and extrinsic motivations, willingness of employees to innovate increases. Furthermore, employees with higher creative self-efficacy may create innovative behaviors for their recognition of “have ability to do.” At the same time, employees perceive a strong organizational innovation reward policy, exerting efforts to achieve continuous innovation. By contrast, when the innovation reward policy of organization is weak, expectations of employees of the consequences of innovative behavior decline. Even if employees with a high sense of creative self-efficacy may undertake a routine work instead of innovative behaviors because innovation cannot bring good returns. Based on this discussion, the sixth hypothesis is proposed.

***Hypothesis 6:****Innovation rewards play a moderating role in the relationship between creative self-efficacy and employee innovative behavior. Compared with the lower innovation rewards, the positive relationship between creative self-efficacy and employee innovation behavior is stronger under the higher innovation rewards*.

### Moderated Mediating Effect

Hypotheses 2 and 3, respectively, explain the mediating role of psychological safety and creative self-efficacy between inclusive leadership and employee innovative behavior. Hypotheses 5 and 6, respectively, illustrate a moderating effect of innovation rewards on the relationship between “psychological safety—employee innovation behavior” and “creative self-efficacy—employee innovation behavior.” According to the above discussions, this study integrates social cognitive and motivation theories to construct a moderating multiple mediation model, which is based on the moderating mediator inference method (Edwards and Lambert, 2007). That is, innovation rewards positively moderate the mediating effects of both psychological safety and of creative self-efficacy on the relationship between inclusive leadership and employee innovative behavior. Thus, the following hypotheses are proposed.

***Hypothesis 7:****Innovation rewards positively mediates the indirect effect of inclusive leadership on employee innovative behavior through psychological safety. That is, the higher the level of innovation rewards, the greater the mediating effect of psychological safety*.
***Hypothesis 8:****Innovation rewards positively mediate the indirect effects of inclusive leadership on employee innovative behavior through creative self-efficacy. That is, the higher the level of innovation rewards, the greater the mediating effect of creative self-efficacy*.

In summary, the theoretical model of this study is shown in Figure 1.

## Materials and Methods

### Sample and Procedures

This study examines the theoretical model using the data collected from the employees of enterprises in the manufacturing industry of China, which is selected for two reasons: China has a massive manufacturing industry and is implementing a strategy of innovation-driven development. Following this strategy, a large number of manufacturing industry enterprises in China are promoting technological change and industrial upgrading through innovation.

Participants are recruited in a following way. Firstly, 20 enterprises that belong to the manufacturing industry are identified through MBA alumni. Secondly, the human resource department directors of these enterprises are contacted and the purpose of data collection is explained. From these 20 enterprises, 484 employees are recruited to participate in the questionnaire survey. Then, a private email is sent to all participants several days before the questionnaire survey to explain the research procedure and to emphasize that the survey is for academic research purposes only and is strictly under complete confidentiality.

The questionnaire survey is composed of two stages: Time 1 (T1), in which employees complete questionnaires regarding a predictor variable (inclusive leadership), a moderating variable (innovation rewards), and demographic variables (age, gender, education, time spent working with the current leader, and department). After a month, at Time 2 (T2), the same participants completed questionnaires regarding mediating variables (psychological safety and creative self-efficacy) and a dependent variable (employee innovative behavior). To match the responses of T1 and T2, participants were asked to fill in the last four digits of their ID numbers in the questionnaire.

A total of 484 questionnaires are finally collected. About 66 are discarded for missing data, leaving 418 valid questionnaires and the response rate of 86.4%. Among the samples, 226 (54.1%) are males and 192 (45.9%) are females. In terms of age, 87 (20.8%) are below 25 years old, 145 (34.7%) are between 25 and 29 years old, 135 (32.3%) are between 30 and 39 years old, 34 (8.1%) are between 40 and 50 years old, and 17 (4.1%) are over 50 years old. In terms of time spent working with the current leader, 63 (15.1%) answered less than 3 years, 193 (46.2%) for 3–5 years, 100 (23.9%) for 6–10 years, and 62 (14.8%) for more than 11 years. In terms of education, 41(9.8%) reach a senior high school degree or below, 103 (24.6%) has a junior college degree, 210 (50.2%) has a bachelor's degree, and 64 (15.3%) has a master's degree or above. In terms of department of employee, the administration department accounts for 18.7% (78), the technology/R&D department accounts for 30.9% (129), the marketing department accounts for 13.4% (56), the finance and accounts department accounts for 6.7% (28), the human resource department accounts for 12.9% (54), the sale department accounts for 4.8% (20), the operation department accounts for 8.6% (38), and the logistic department accounts for 4.1% (17).

### Measures

All item scales are originally developed in English and are therefore translated into Chinese, and all parameters are measured on a five-point Likert scale from 1 = “strongly disagree” to 5 = “strongly agree.”

### Inclusive Leadership

Inclusive leadership is measured with the nine-item scale developed by Carmeli et al. (2010). The items are as follows: (1) my leader is open to hearing new ideas; (2) my leader is attentive to new opportunities to improve work processes; (3) my leader is open to discuss the desired goals and new ways to achieve them; (4) my leader is available for consultation on problems; (5) my leader is an ongoing “presence” in this team—someone who is readily available; (6) my leader is available for professional questions I would like to consult with him/her; (7) my leader is ready to listen to my requests; (8) my leader encourages me to access him/her on emerging issues; and (9) my leader is accessible for discussing emerging problems. Cronbach's alpha for this scale is 0.916.

### Psychological Safety

Psychological safety is measured with the five-item scale developed by Liang et al. (2012). The items are as follows: (1) in my work unit, I can express my true feelings regarding my job; (2) in my work unit, I can freely express my thoughts; (3) in my work unit, expressing your true feelings is welcomed; (4) nobody in my unit will pick on me even if I have different opinions; and (5) I am worried that expressing true thoughts in my workplace would do harm to myself (reverse-coded). Cronbach's alpha for this scale is 0.844.

### Creative Self-Efficacy

Creative self-efficacy is measured with the 13-item scale developed by Yang and Cheng (2009). Considering repetition in the translation, three items are deleted and the scale with 10 items is used. The items are as follows: (1) the belief that I would suggest new ways to achieve goal or objectives; (2) the belief that I would come up with new and practical ideas to improve performance; (3) the belief that I could search out new technologies, processes, techniques, and/or product ideas; (4) the belief that I would be a good source of creative ideas; (5) the belief that I would be not afraid to take risks; (6) the belief that I would promote and champion ideas to others; (7) the belief that I would exhibit creativity on the job when given the opportunity too; (8) the belief that I would develop adequate plans and schedules for the implementation of new ideas; (9) the belief that I would often come up with creative solutions to problems; and (10) the belief that I would suggest new ways of performing work tasks. Cronbach's alpha for this scale is 0.913.

### Innovation Rewards

Innovation reward is measured with the eight-item scale developed by Yoon and Choi (2010). The items were as follows: (1) when I perform creatively, I receive financial rewards, such as incentives or bonuses; (2) when I perform creative work, it influences my promotion; (3) if I suggest new ideas for tasks, this approach influences my performance evaluation; (4) I am recognized by my supervisor when I suggest new ideas for the task; (5) my coworkers recognize me when I perform creatively at work; (6) when an employee exhibits creative performance, my company offers several treats such as a celebration dinner; (7) when I perform creatively at work, my company offers corresponding benefits in return; and (8) when I perform creatively at work, my manager or the top management compliments me publicly. Cronbach's alpha for this scale is 0.897.

### Employee Innovative Behavior

Employee innovative behavior is measured using the six-item scale developed by Scott and Bruce (1994). The items are as follows: (1) searches new technologies, processes, techniques, and/or product ideas; (2) generates creative ideas; (3) promotes and champions ideas to others; (4) investigates and secures funds needed to implement new ideas; (5) develops adequate plans and schedules for the implementation of new ideas; and (6) in general, I am an innovative person. We chose the self-report measure of employee creativity for the following reasons: firstly, creativity usually begins in an “awareness” stage during which individuals both recognize an opportunity to be creative and formulate a potential innovation (Ong et al., 2003). Secondly, employees may be better able than supervisors and peers to judge the extent to which new ideas are fundamentally or incrementally creative within the work context (Ng and Feldman, 2012). Finally, the employee innovative behavior scale developed by Scott and Bruce (1994) was usually measured by self-ratings (Carmeli et al., 2010; Xerri, 2014; Hsu and Chen, 2015; Purc and Laguna, 2017). Thus, the self-report scale is chosen to measure employee innovative behavior. Cronbach's alpha for this scale is 0.911.

### Control Variables

Previous literature studies have shown that demographic variables may influence employee innovative behavior including age, gender, education, department, and time spent working with the current leader (e.g., Carmeli et al., 2010; Javed et al., 2017, 2020; Fang et al., 2019; Su et al., 2019; Wang et al., 2019). Thus, gender, age, education, department, and time spent working with the current leader were chosen as control variables in this study. Gender is measured as a dummy variable (1 = male, 2 = female). Age is divided into five levels (1 = under 25 years, 2 = 25–29 years, 3 = 30–39 years, 4 = 40–50 years, 5 = over 50 years). Education is divided into four levels (1 = senior high school or below, 2 = junior college, 3 = bachelor, 4 = postgraduate). Time spent working with current leader is divided into four levels (1 = less than 3 years, 2 = 3–5 years, 3 = 6–10 years, 4 = more than 11 years). Department of employee is divided into eight levels (1 = administration, 2 = technology/R&D, 3 = marketing, 4 = finance and accounting, 5 = human resource, 6 = sale, 7 = operations, 8 = logistics).

### Data Analysis

The statistical software SPSS 25.0 and Mplus 7.4 were used for data analysis. Firstly, SPSS 25.0 was used to test the reliability of the five key variables in this study. Secondly, Mplus 7.4 was used for a confirmatory factor analysis (CFM) to test the discriminant validity and common method variance (CMV). Thirdly, SPSS 25.0 was used for descriptive statistics and correlation analysis. Fourthly, structural equation modeling (SEM) was used to examine the theoretical model. Finally, regression and bias-corrected bootstrapping analyses were used to test the hypotheses.

## Results

### Discriminant Validity

In the aspect of validity test, firstly, the average variance extracted (AVE) of inclusive leadership, employee innovative behavior, psychological safety, creative self-efficacy, and innovation rewards, respectively, were 0.550, 0.632, 0.521, 0.512, and 0.532, all of which were greater than the critical value 0.5 and squared correlations between variables (Fornell and Larcker, 1981). The results indicated that the questionnaire has good convergent validity. Secondly, MPLUS7.4 was used to carry out the CFA. Compared with other competition models, the theoretical five-factor model (inclusive leadership, employee innovative behavior, psychological safety, creative self-efficacy, and innovation rewards) had a better fit to the data [χ^2^*/df* = 1.794, comparative fit index (CFI) = 0.938, Tucker–Lewis index (TLI) = 0.934, root mean squared error of approximation (RMSEA) = 0.044, standardized root mean square residual (SRMR) = 0.041] (see Table 1). The results of CFA showed that the theoretical five-factor model had satisfactory discriminant validity.

**Table 1:** Results of confirmatory factor analyses.

	**Factors**	***χ^2^***	***df***	***χ^2^/df***	**RMSEA**	**TLI**	**CFI**	**SRMR**
Five-factor model	IL,PS,CSE,IR,EIB	1,174.997	655	1.794	0.044	0.934	0.938	0.041
Four-factor model	IL, PS+CSE, IR, EIB	2,695.950	659	4.091	0.086	0.743	0.759	0.110
Three-factor model	IL, PS+CSE+IR, EIB	3,341.000	662	5.047	0.098	0.663	0.683	0.121
Two-factor model	IL+PS+CSE+IR, EIB	4,909.415	664	7.394	0.124	0.468	0.497	0.146
One-factor model	IL+PS+CSE+IR+EIB	5,886.375	665	8.85	0.137	0.346	0.381	0.153
Unmeasured latent method factor model		1,174.995	654	1.797	0.044	0.934	0.938	0.041

*IL, inclusive leadership; PS, psychological safety; CSE, creative self-efficacy; IR, innovation rewards; EIB, employee innovative behavior*.

### Common Method Variance

Although the anonymous measurement method and two-wave design in a survey were used to reduce CMV in the data collection. However, CMV may still occur because all variables were measured by individual self-evaluation, and all items in each questionnaire were provided by the same survey object. Thus, the Harman single-factor test was used to assess the existence of CMV. The results showed that the first factor solution in the exploratory factor analysis indicated only explained 24.652% (<50%) loading, which proved the absence of CMV (Woszczynski and Whitman, 2004). Further, we conducted the unmeasured latent method factor, that all items were loaded on both this latent method factor and trait factors (Podsakoff et al., 2003), to test CMV. A comparison of the latent method factor model (χ^2^*/df* = 1.797, CFI = 0.938, TLI = 0.934, RMSEA = 0.044, SRMR = 0.041) and the theoretical five-factor model (χ^2^*/df* = 1.794, CFI = 0.938, TLI = 0.934, RMSEA = 0.044, SRMR = 0.041) indicated no changes in CFI (Cheung and Rensvold, 2002). Thus, CMV should not be a severe problem in our study.

### Descriptive Statistics and Correlation Analysis

Table 2 shows the results of descriptive statistics (mean and SD) and correlation analysis (Pearson coefficient). Inclusive leadership is positively correlated to employee innovative behavior (*r* = 0.380, *p* < 0.001), psychological safety (*r* = 0.196, *p* < 0.001), and creative self-efficacy (*r* = 0.329, *p* < 0.001). Psychological safety is positively correlated to employee innovative behavior (*r* = 0.304, *p* < 0.001). Creative self-efficacy is positively correlated to employee innovative behavior (*r* = 0.397, *p* < 0.001). Innovation rewards are positively correlated to employee innovative behavior (*r* = 0.149, *p* < 0.01). The correlation between the key variables provides the initial support for the hypotheses. At the same time, control variables have no significant influence on the key variables, and thus these control variables are not introduced into the subsequent analysis.

**Table 2:** Means, standard deviations, and correlations.

**Variables**	**Mean**	**SD**	**Sex**	**Age**	**Education**	**TSWCL**	**Department**	**IL**	**PS**	**CSE**	**IR**	**EIB**
Gender	1.46	0.499	1									
Age	2.4	1.032	−0.055	1								
Education	2.71	0.842	0.026	−0.074	1							
TSWCL	2.61	0.916	0.017	0.207^***^	−0.026	1						
Department	3.33	2.089	0.064	0.030	0.059	−0.001	1					
IL	3.238	0.936	0.033	0.002	0.033	−0.027	−0.007	1				
PS	3.157	0.951	−0.038	0.112	0.026	−0.046	−0.013	0.196^***^	1			
CSE	3.251	0.887	−0.053	−0.013	−0.024	−0.092	−0.076	0.329^***^	0.322^***^	1		
IR	3.212	0.907	0.018	−0.023	−0.112	−0.081	−0.100	−0.027	−0.025	−0.049	1	
EIB	3.164	1.089	−0.010	0.017	−0.057	−0.107	−0.025	0.380^***^	0.304^***^	0.397^***^	0.149^**^	1

*N = 418;*

** *p < 0.01*,

*** *p < 0.001. TSWCL, time spent working with current leader*.

### Test of Direct Effect and Mediating Effect

**Figure 2:**
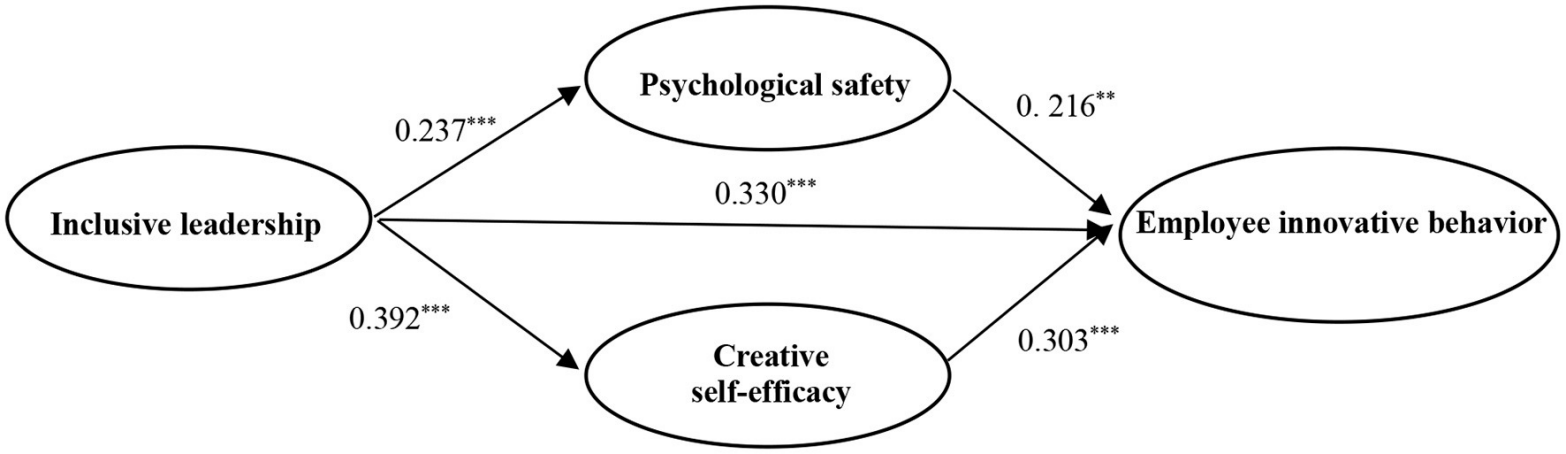
Results of theoretical model by using Mplus. *N* = 418, **p < 0.01, ^***^p < 0.001. Standardized path coefficients are reported.

Given that the theoretical model is a multiple mediator model, MPLUS 7.4 is used to construct a structural equation model, where the bootstrap method based on deviation correction is used to test the multiple mediation effects. The sample size is set to 5,000, and 95% CI is obtained. As given in Table 3, the results of the multiple mediator model (inclusive leadership, employee innovative behavior, psychological safety, and creative self-efficacy) have a good fit (χ^2^*/df* = 1.876, CFI = 0.948, TLI = 0.943, RMSEA = 0.046, SR= 0.061), and further analysis can be performed. Figure 2 shows the results of SEM with the standardized coefficients. Table 3 also presents the results of the bootstrap test of deviation correction. The direct and mediating effects are confirmed in Figure 2 and Table 3.

**Table 3:** Results of multiple mediating effects test.

**Effects**	**Estimate**	**S.E**.	**Est./S.E**.	***P*-value**	**95% CI**	
					**Lower limit**	**Upper limit**
Total effect IL → EIB	0.500	0.066	7.519	0.000	0.390	0.609
Direct effect IL → EIB	0.330	0.067	4.938	0.000	0.220	0.440
Direct effect IL → PS	0.237	0.056	4.207	0.000	0.144	0.329
Direct effect PS → EIB	0.216	0.063	3.444	0.001	0.113	0.319
Direct effect IL → CSE	0.392	0.065	6.079	0.000	0.286	0.498
Direct effect CSE → EIB	0.303	0.065	4.679	0.000	0.196	0.409
Indirect effect IL → PS → EIB	0.051	0.019	2.642	0.008	0.019	0.083
Indirect effect IL → CSE → EIB	0.119	0.031	3.889	0.000	0.068	0.169
IND1+IND2	0.170	0.038	4.450	0.000	0.107	0.232
IND1–IND2	−0.068	0,034	−1.988	0.047	−0.123	−0.012
Goodness of fit test				*χ^2^/df* = 1.876 CFI = 0.948 TLI = 0.943 RMSEA = 0.046 SRMR = 0.061		

*IL, inclusive leadership; PS, psychological safety; CSE, creative self-efficacy; EIB, employee innovative behavior. IND1–IND2, the mediating effect of psychological safety subtract the mediating effect of creative self-efficacy; IND1+IND2, the mediating effect of psychological safety plus the mediating effect of creative self-efficacy*.

#### Direct Effect Test

In Figure 2, the direct effect of inclusive leadership on employee innovative behavior is supported by the regression coefficient and associated significance level (β = 0.330, *p* < 0.001). Furthermore, in Table 3, the total effect coefficient of inclusive leadership on employee innovative behavior is significant (β = 0.500, *p* < 0.001), and the 95% CI is [0.390, 0.609]. The results suggest that inclusive leadership is significantly positively related to employee innovative behavior. Thus, Hypothesis 1 is confirmed.

#### Mediating Effect Test

In Figure 2, inclusive leadership is positively related to psychological safety (β = 0.237, *p* < 0.001), and psychological safety is positively related to employee innovative behavior (β = 0.216, *p* < 0.01). Furthermore, in Table 3, after controlling creative self-efficacy, the indirect effect of “IL→PS→ EIB” is significant (β = 0.051, *p* < 0.01), the CI is [0.019, 0.083] (excluding zero). The results suggest that psychological safety plays a mediating role between inclusive leadership and employee innovative behavior. Thus, Hypothesis 2 is confirmed.

Figure 2 shows that inclusive leadership is positively related to creative self-efficacy (β = 0.392, *p* < 0.001), and creative self-efficacy is positively related to employee innovative behavior (β = 0.303, *p* < 0.001). Furthermore, in Table 3, after controlling psychological safety, the indirect effect of “IL→CSE→EIB” is significant (β = 0.119, *p* < 0.001), and the CI is [0.068, 0.169] (excluding zero). The results suggest that creative self-efficacy plays a mediating role between inclusive leadership and employee innovative behavior. Thus, Hypothesis 3 is confirmed.

#### Comparison of Mediating Effects

As seen in Table 3, the mediating effect coefficient of psychological safety (β = 0.051, *p* < 0.01) is smaller than that of creative self-efficacy (β = 0.119, *p* < 0.001). The difference in coefficient between the two mediating effects is significant (β = −0.068, *p* < 0.05), and the CI is [−0.123, −0.012] (excluding zero). That is to say, the mediating effect of psychological safety is less than that of creative self-efficacy.

### Test of Moderating Effect and Moderated Mediating Effect

**Figure 3:**
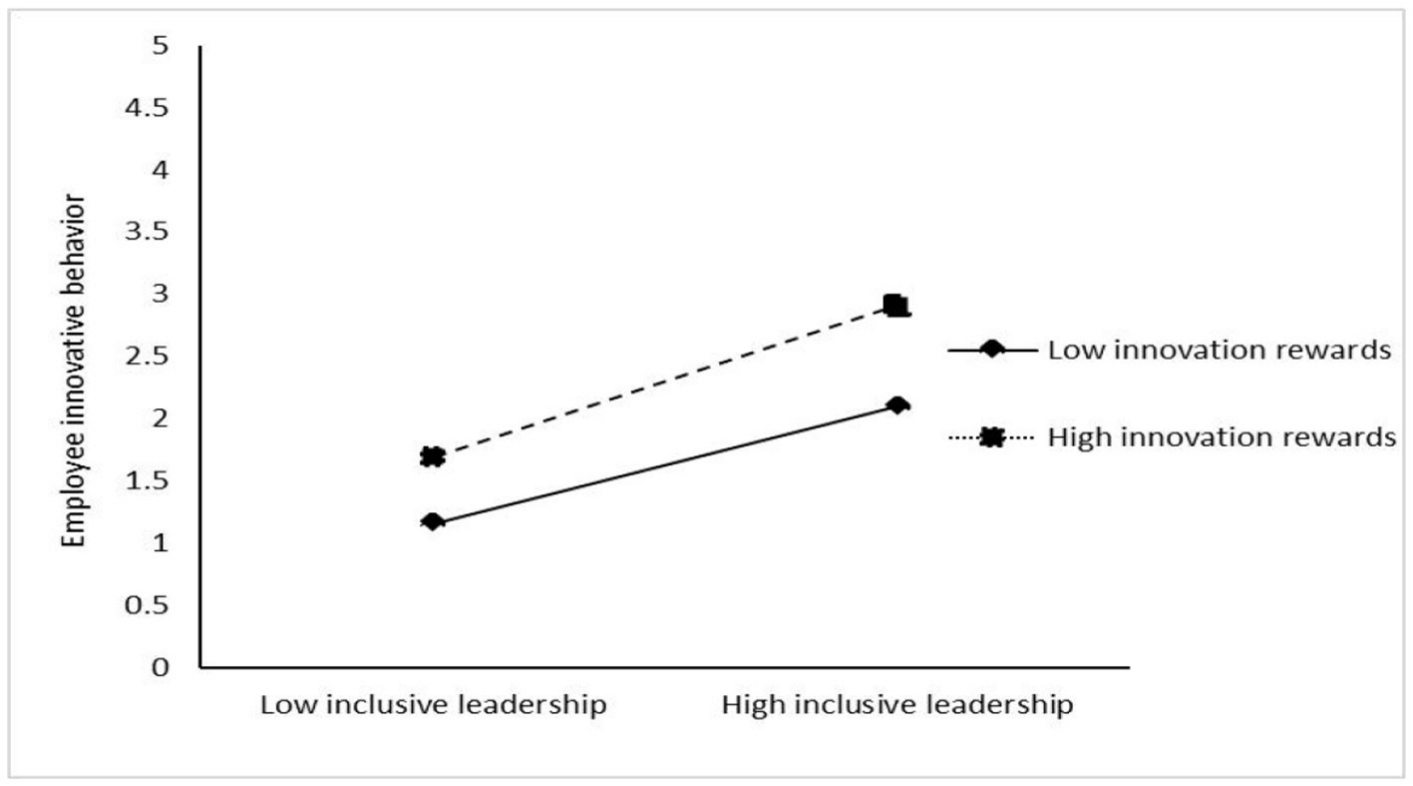
Moderating effect of innovation rewards on the relationship of inclusive leadership and employee innovative behavior.

**Figure 4:**
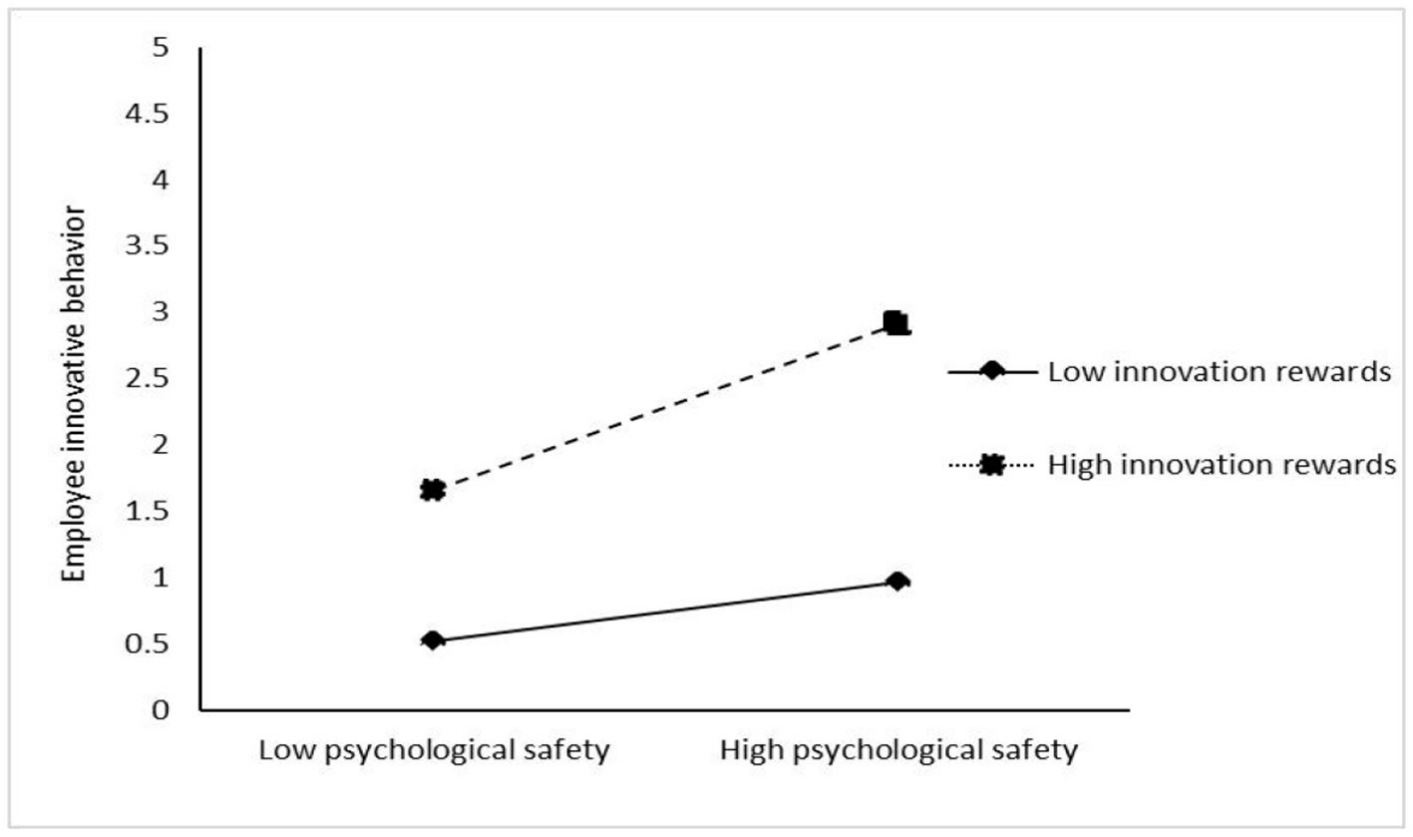
Moderating effect of innovation rewards on the relationship of psychological safety and employee innovative behavior.

**Figure 5:**
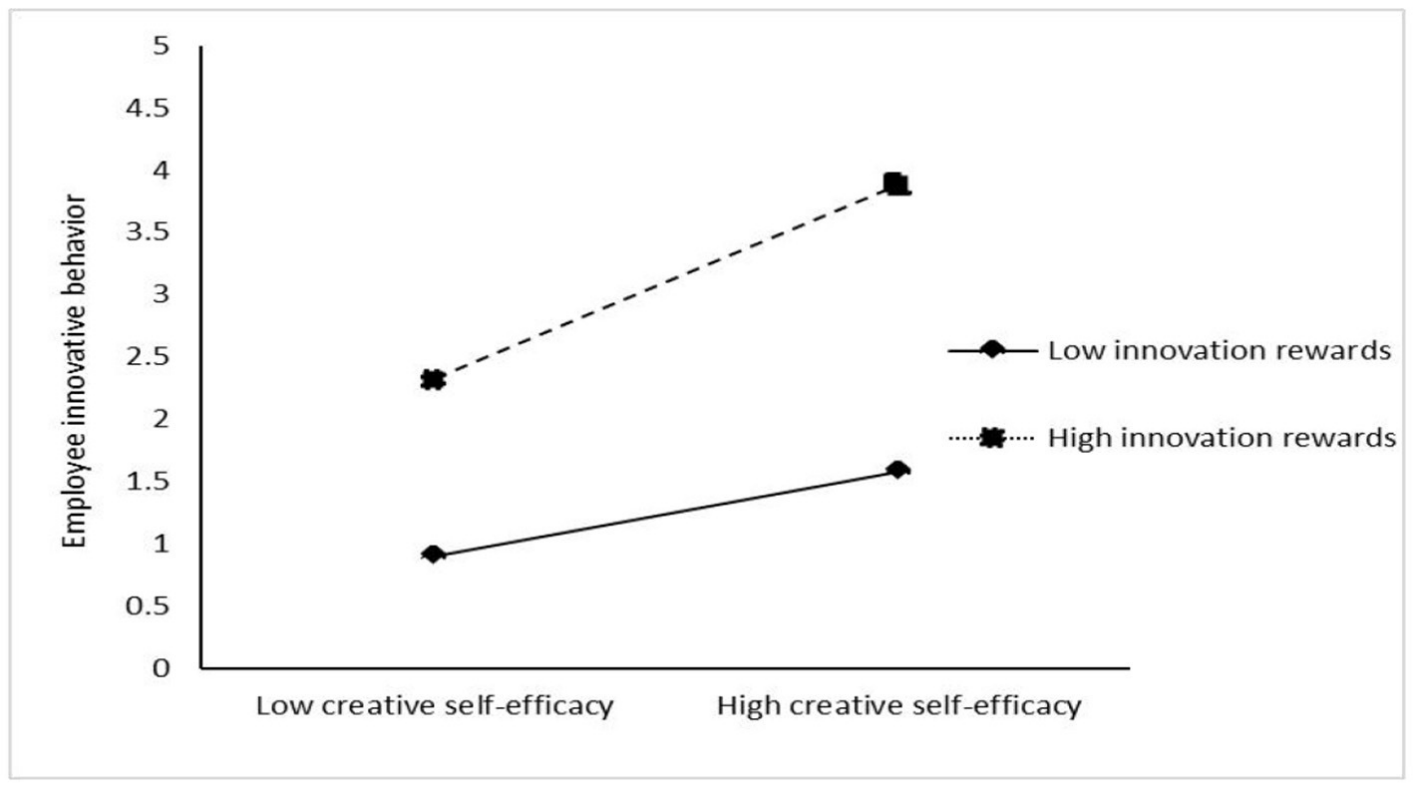
Moderating effect of innovation rewards on the relationship of creative self-efficacy and employee innovative behavior.

Table 4 shows the results of the test for a moderating effect. The interaction of inclusive leadership and innovation rewards is significantly and positively related to employee innovative behavior (β = 0.147, *p* < 0.05), showing that innovation rewards positively moderate the relationship of inclusive leadership and employee innovative behavior. The interaction of psychological safety and innovation rewards is significantly and positively related to employee innovative behavior (β = 0.427, *p* < 0.001), showing that innovation rewards positively moderate the relationship between psychological safety and employee innovative behavior. Meanwhile, the interaction of self-efficacy and innovation reward is significantly and positively related to employee innovative behavior (β = 0.503, *p* < 0.001), showing that innovation rewards play a positive role in moderating the relationship between creative self-efficacy and employee innovative behavior. In addition, to clearly show the moderating effect of innovation rewards, this study adds and subtracts one SD from the mean value of innovation rewards and constructs two groups of high and low innovation rewards. Then, the regressions, respectively, are calculated in the regression equation, and the adjustment effect diagram is drawn according to the regression coefficient (Figures 3–5). Figure 3 shows that compared with the low level of innovation rewards, the inclusive leadership has a greater impact on employee innovative behavior under the high level of innovation rewards. Thus, innovation rewards positively moderates the impact of inclusive leadership on employee innovation behavior, supporting Hypothesis 4. Figure 4 shows that compared with the low level of innovation rewards, the psychological safety has a greater impact on employee innovative behavior under the high level of innovation rewards. Therefore, the results support Hypothesis 5. Figure 5 shows that compared with the low level of innovation rewards, self-efficacy has a greater impact on employee innovative behavior under the high level of innovation rewards. Thus, the results support Hypothesis 6.

**Table 4:** Results of moderating effect test.

**Effects**	**Estimate**	**S.E**.	**Est./S.E**.	***P*-value**
IL × RFI → EIB	0.147	0.067	2.210	0.027
PS × RFI → EIB	0.427	0.064	6.625	0.000
SE × RFI → EIB	0.503	0.051	9.841	0.000

For the moderated mediating effect test, a “mediating effect difference test” is used. By adding or subtracting one SD from the mean value of innovation rewards, the conditional mediating effects of psychological safety and creative self-efficacy under high and low innovation rewards are formed and compared for significance at different levels. If the CI excludes zero, the mediated mediating effect is significant. Table 5 shows the results. The model of inclusive leadership influencing employee innovative behavior through psychological safety shows that at low levels of innovation rewards, the mediating effect of psychological safety is significant (β = 0.323, *p* < 0.001, the CI is [0.178, 0.482], excluding zero). At high levels of innovation rewards, the mediating effect of psychological safety is significant (β = 0.528, *p* < 0.001, the CI is [0.292, 0.787], excluding zero). The two groups show significant differences (β = 0.205, *p* < 0.001, the CI was [0.114, 0.305], excluding zero). The results suggest that innovation rewards positively moderate the mediating effect of psychological safety. Thus, Hypothesis 7 is confirmed.

**Table 5:** Results of moderated mediating effect test.

**Effects**	**Estimate**	**S.E**.	**Est./S.E**.	***P*-value**	**95% CI**	
					**Lower limit**	**Upper limit**
**Inclusive leadership—Psychological safety—Employee innovative behavior**						
Low innovation rewards (−1SD)	0.323	0.093	3.489	0.000	0.178	0.482
High innovation rewards (+1SD)	0.528	0.150	3.521	0.000	0.292	0.787
Differences between the two groups	0.205	0.058	3.525	0.000	0.114	0.305
**Inclusive leadership—Creative self-efficacy—Employee innovative behavior**						
Low innovation rewards (−1SD)	0.480	0.082	5.819	0.000	0.346	0.624
High innovation rewards (+1SD)	0.764	0.134	5.696	0.000	0.547	0.998
Differences between the two groups	0.284	0.053	5.404	0.000	0.201	0.375

*The difference between the two groups is equal to the mediating effect of conditions under high innovation rewards minus the mediating effect of conditions under low innovation rewards*.

The model of inclusive leadership influencing employee innovative behavior through creative self-efficacy shows that at low levels of innovation rewards, the mediating effect of creative self-efficacy is significant (β = 0.480, *p* < 0.001, the CI is [0.346, 0.624], excluding zero). At high levels of innovation rewards, the mediating effect of creative self-efficacy is significant (β = 0.764, *p* < 0.001, the CI is [0.547, 0.998], excluding zero). The two groups show significant differences (β = 0.284, *p* < 0.001, the CI was [0.201, 0.375], excluding zero). The results suggest that innovation rewards positively moderate the mediating effect of creative self-efficacy. Therefore, Hypothesis 8 is confirmed.

## Discussion

The past few years have witnessed a growing academic interest in the relationship between inclusive leadership and employee innovative behavior (e.g., Carmeli et al., 2010; Javed et al., 2017, 2018; Fang et al., 2019; Wang et al., 2019). The present study takes a step further to explore the employee innovative behavior outcomes of inclusive leadership. Based on the social cognitive and motivation theories, with psychological safety and creative self-efficacy as mediating variables and innovation rewards as moderating variables in the second half, this study constructs a moderating multiple mediation model. Consistent with the hypotheses, the theory model is confirmed by empirical research. The conclusions are as follows.

Firstly, inclusive leadership positively affects employee innovative behavior; the more inclusive leadership behavior shown by the leader, the more effective it is in stimulating employee innovative behavior. This finding agrees with previous studies on the concept that inclusive leadership is a primary factor behind the promotion of employee innovative behavior (e.g., Carmeli et al., 2010; Javed et al., 2017, 2018; Fang et al., 2019; Wang et al., 2019).

Secondly, psychological safety partially mediates the relationship between inclusive leadership and employee innovative behavior. When enterprise leaders show inclusive leadership, the psychological safety of employees improves, which stimulates their innovative behavior. This finding supports the assumption that psychological safety mediates the relationship between inclusive leadership and employee innovative behavior (Carmeli et al., 2010; Javed et al., 2017; Zhu et al., 2019; Mansoor et al., 2020). However, different from previous studies, the present findings also show that creative self-efficacy partially mediates the impact of inclusive leadership on employee innovative behavior. When the enterprise leaders show inclusive leadership, the creative self-efficacy of employees improves, which stimulates their innovative behavior. This finding supports the notion that creative self-efficacy mediates the relationship between inclusive leadership and innovative behavior (Javed et al., 2020). Moreover, the findings extend the social cognitive theory by providing the empirical evidence of the mediating effect of both psychological safety and creative self-efficacy on the relationship of inclusive leadership and employee innovative behavior.

Thirdly, the mediating effect of psychological safety is significantly weaker than that of creative self-efficacy. This is an interesting finding and can be interpreted in a way that employee innovative behavior is full of risks and challenges. In exhibiting innovative behavior, an individual considers both “Do I have ability to do?” and “Is it safe to do?”; but as the proverb says, “They can do it because they think they can.” Compared with psychological safety, creative self-efficacy is more important for employee innovative behavior. Previous studies proved that both psychological safety and creative self-efficacy mediate the relationship of inclusive leadership and employee innovative behavior. However, most studies emphasize that psychological safety is an important mediating role between the relationship as mentioned earlier (Carmeli et al., 2010; Javed et al., 2017; Zhu et al., 2019; Mansoor et al., 2020). Furthermore, a comparative analysis on the mediating effect of psychological safety and that of creative self-efficacy is lacking. The present finding extends the mediation mechanism of inclusive leadership on employee innovative behavior.

Fifthly, innovation rewards positively moderate the relationship of inclusive leadership and employee innovative behavior. Compared with the low innovation rewards, the positive relationship between inclusive leadership and employee innovative behavior is stronger under high innovation rewards. Furthermore, innovation rewards positively moderate the effect of psychological safety on employee innovative behavior. That is, at high levels of innovation rewards in enterprises, there is a strong positive effect of psychological safety on employee innovative behavior. By contrast, at low levels of innovation rewards, there is a weak positive effect of psychological safety on employee innovative behavior. Meanwhile, innovation rewards positively moderate the effect of creative self-efficacy on employee innovative behavior. That is, at high levels of innovation rewards in enterprises, there is a strong positive effect of creative self-efficacy on employee innovative behavior. By contrast, at low levels of innovation rewards, there is a weak positive effect of creative self-efficacy on employee innovative behavior. Furthermore, innovation rewards positively moderate the mediating role of psychological safety and creative self-efficacy, that is, compared with low-level innovation rewards, psychological safety and creative self-efficacy have a strong mediating effect under high-level innovation rewards. These findings agree with the arguments that innovation is not only a cognitive activity but also a behavior driven by motivation (Jong and Hartog, 2007).

### Theoretical Implications

Firstly, this study extends the current understanding of the mediating mechanism of inclusive leadership on employee innovative behavior. According to the theory of social cognition, this study integrates psychological safety and creative self-efficacy into the same analytical framework to explore the mechanism of inclusive leadership on employee innovative behavior. Although the mediating effect of psychological safety between the relationship of inclusive leadership and employee innovative behavior has been explained (Carmeli et al., 2010; Javed et al., 2017; Zhu et al., 2019; Mansoor et al., 2020), previous research mainly rely on the leader–member exchange theory that focuses on the relational mechanism. Moreover, to date, limited research has attended to explore the mediating effect of creative self-efficacy between the relationship of inclusive leadership and employee innovative behavior (Javed et al., 2020). This study responds to this call by investigating the role of creative self-efficacy as a mediating mechanism in the relationship between inclusive leadership and employee innovative behavior (Javed et al., 2017). The findings suggest that inclusive leadership not only indirectly affects employee innovative behavior through psychological safety but also through creative self-efficacy. Furthermore, the focus on the cognitive mechanism thus broadens the existing knowledge of the mediating effect of psychological safety between inclusive leadership and employee innovative behavior.

Secondly, this study compares the different mediating mechanisms of inclusive leadership on employee innovative behavior. Previous research on the relationship of inclusive leadership and employee innovative behavior was mainly focused on different mediating variables, such as psychological safety (Carmeli et al., 2010; Javed et al., 2017; Zhu et al., 2019; Mansoor et al., 2020), leader–member exchange (Javed et al., 2018), psychological empowerment (Randel et al., 2017; Javed et al., 2018), and organizational commitment (Choi et al., 2015). However, a comparative analysis of different mediating mechanisms is lacking. With an aim to fill this gap, this study constructs a dual mediation model, which integrates psychological safety and creative self-efficacy into the same research framework, such that these two different mediating effects can be compared. The findings suggest that the mediating effect of psychological safety is significantly less than that of creative self-efficacy. Previous research had been unanimous on the importance of the mediating mechanism of psychological safety, whereas a few studies endeavored to uncover the mediating mechanism of creative self-efficacy. The present findings call for attention to creative self-efficacy.

Thirdly, this study extends the research of the boundary conditions under which the mediating effect of psychological safety and creative self-efficacy are strong or weak. To our knowledge, no existing study has attempted to explore the moderating mechanism of inclusive leadership's mediating effect on creativity. This study is a step toward filling this gap using innovation rewards as a moderating variable. According to the motivation theory, motivation is an important psychological motivation that determines the form, direction, intensity, and duration of behavior. As demonstrated in previous studies, innovation rewards can inspire employee motivation to lead to more innovative behaviors (Byron and Khazanchi, 2012; Malik et al., 2015). This study explores the moderating role of innovation rewards in the relationship of inclusive leadership and employee innovative behavior based on the motivation theory. Furthermore, this study integrates social cognition and motivation theories to explore the moderating mechanism of innovation rewards. The findings show that innovation rewards not only play a positive moderating role in the relationship between creative self-efficacy and employee innovative behavior but also play a positive moderating role in the relationship between psychological safety and employee innovative behavior. This finding is in agreement with the argument that to achieve continuous innovation, employees should be both able and willing to innovate (Jong and Hartog, 2007). Innovation rewards provide benefits for employees who carry out innovative behaviors. This positive reinforcement can stimulate the motivation of employees for innovation. The interaction of psychological safety and innovation rewards provides employees with both the capacity and willingness to innovate. Meanwhile, the interaction of creative self-efficacy and innovation rewards also provides employees with “have the ability to do” cognition and “have reasons to do” motivation, which positively affect employee innovative behavior. Thus, the findings extend the moderating mechanism between inclusive leadership and employee innovative behavior and enriches the studies on inclusive leadership.

Fourthly, this study extends the literature on social cognitive and motivation theories through their integration to explore the mediating mechanism of both psychological safety and creative self-efficacy and the moderating mechanism of innovation rewards. Although innovation is not only a cognitive activity but also a behavior driven by motivation (Jong and Hartog, 2007), most previous studies are only based on a perspective of cognition or motivation. The present study integrates two different perspectives and makes up for the deficiency of previous studies on the influence of cognition–motivation interaction on innovative behavior. Thus, the influence of the mechanism of employee innovative behavior is further explored.

Finally, this study expands the research on inclusive leadership and employee innovative behavior in a distinct cultural and national context. This study is conducted in China, which has notable differences to that of western contexts. Nembhard and Edmondson (2006) originally proposed the concept of inclusive leadership in the field of management, and defined this as the “words and deeds by a leader or leaders that indicate an invitation and appreciation for the contribution from others.” However, research on inclusive leadership in the Chinese context has been scare (Fang et al., 2019; Qi et al., 2019; Wang et al., 2019; Ye et al., 2019). The Chinese culture advocates values such as “harmony without uniformity and inclusiveness” and “tolerance is a virtue,” which contains the idea of “inclusiveness.” Thus, research on inclusive leadership in the Chinese context is of considerable significance. This study expands inclusive leadership and employee innovative behavior research in a different cultural and national context.

### Practical Implications

At present, the environmental complexity due to new technological changes has made it vital for organizations to be innovative in products, services or work process, and organization innovations rely on employee innovative behavior, so it is crucial to stimulate the innovative behavior of employees. This study explores the impact mechanism of inclusive leadership on employee innovative behavior and provides practical implications for enterprise innovation management.

The findings show that inclusive leadership has a positive effect on employee innovative behavior. Based on these findings, this study recommends that organizations can take active measures to promote inclusive leadership through the human resource management practices. Firstly, organizations can adopt a practice of prioritizing the hiring of managers who possess inclusive attributes such as openness, availability, and accessibility (Javed et al., 2020). Secondly, organizations can promote the inclusive behavior of managers through leadership training. Thirdly, organizations can take incentive measures to encourage and motivate managers to implement more inclusive behaviors, for example, considering the inclusive behavior of manager in a performance appraisal system through the subordinate's evaluation of their superior. Fifthly, organizations can create an inclusive atmosphere in the construction of corporate culture. Moreover, this study recommends that managers can bring their inclusive leadership style through the following ways. Firstly, managers can show respect to their subordinates and identify and praise the contribution of the subordinates. Secondly, managers can understand the different needs of subordinates by listening to them attentively. Thirdly, managers can provide timely and constructive feedback to the subordinates. Finally, managers can empower the subordinates to independently decide their work activities and show their trust on the subordinates.

The findings show that psychological safety plays a mediating role in the relationship between inclusive leadership and employee innovative behavior. Based on these findings, this study recommends that managers can exhibit inclusive behaviors in the work to encourage employee innovative behavior by enhancing their psychological safety. Given that different employees have different needs, managers can show respect to their subordinates and accept the differences of the subordinates, so as to enhance their psychological safety. Moreover, generating new ideas is a trial and error process, where some of the new ideas generated by employees are likely to fail. Managers can tolerate the failure of their subordinates and give them encouragement and resource support so as to enhance their psychological safety. In addition, organizations must pay attention to enhance the psychological safety of employees. On one hand, organizations can establish a good communication platform to provide convenient channels for employees to express their ideas to enhance their psychological safety. On the other hand, organizations can encourage employees to exhibit innovative behavior by creating an inclusive atmosphere.

The findings show that creative self-efficacy also plays a mediating role in the relationship between inclusive leadership and employee innovative behavior, and the mediating effect of creative self-efficacy is significantly greater than that of psychological safety. Based on these findings, this study suggests that organizations and managers must pay more attention to enhance the creative self-efficacy of employees. On one hand, managers must exhibit inclusive behaviors to encourage employee innovative behavior by enhancing their creative self-efficacy, for example, providing employees autonomy in their activities related to their particular job (Javed et al., 2020), and providing guidance and assistance when employees encounter difficulties (Ye et al., 2018). On the other hand, as creative self-efficacy is an individual recognition of his/her own ability, organizations can establish a training system to improve the skill and ability of employees, which can enhance their creative self-efficacy.

The findings show that innovation rewards play not only a positive moderating role in the relationship between creative self-efficacy and employee innovative behavior but also a positive moderating role in the relationship between psychological safety and employee innovative behavior. Based on these findings, this study recommends that organizations must implement a more complex innovation reward system, which includes both monetary rewards and non-monetary rewards. Firstly, organizations can consider more complex monetary incentives schemes than simply offering separate monetary rewards for employee innovative behavior (Ihl et al., 2019). Secondly, an organization that decides to incentive employee innovative behavior *via* new rules for distributing monetary reward needs to previously assess the size of the reward that is required to make most employees to engage in innovative behaviors (Navaresse et al., 2014; Andreeva et al., 2017). Thirdly, organizations can consider non-monetary rewards such as the form of symbolic public recognition, individual praise, and promotion opportunities (Fischer et al., 2019).

Finally, this study is conducted in China, and Chinese traditional culture advocates values such as “harmony without uniformity and inclusiveness” and “tolerance is a virtue.” Managers with higher traditional values may have more inclusive behaviors. This study confirms the positive effect of inclusive leadership on employee innovation behavior in the context of China. Thus, in China, managers in organizations can pay more attention to Chinese traditional culture, and understand, learn, and promote inclusive behavior at work.

## Limitations and Future Research

From the perspective of cognition–motivation integration, this study explores the promoting effect of inclusive leadership on employee innovative behavior and its black box. Although some theoretical contributions have been made, several limitations still remain.

Firstly, consistent with previous research, creative self-efficacy (Tierney and Farmer, 2002; Yang and Cheng, 2009; Su et al., 2019) and innovative behavior (Carmeli et al., 2010; Xerri, 2014; Hsu and Chen, 2015; Purc and Laguna, 2017) of an employee are still measured using the self-perception of a participant in this study. However, self-evaluation of creative self-efficacy and innovative behavior could be affected by biases and underestimation (or overestimation). As respondents may try to maintain cognitive or attitudinal consistency in their responses to survey items about the self-ratings of creative self-efficacy and innovative behavior, thereby giving rise to artificially high correlations. Moreover, as being innovative is often encouraged and rewarded in the workplace (Scott and Bruce, 1994), respondents might believe that reporting higher self-ratings of innovative behavior will raise their status in the eyes of outside observers. Although the anonymous measurement method and two-wave design in the survey were used to reduce CMV in this study, we further encourage future researches to take multiple steps to reduce the threat of a CMV. Firstly, researchers can collect the data on employee innovative behavior from multiple sources (e.g., employees and their superiors). For instance, employees evaluate their creative self-efficacy, whereas their respective supervisors evaluate employee innovative behavior. Further, combining self-ratings with the non-self-report measures of employee innovative behavior to provide an overall index is also justifiable if there is a convergence between the two measures (e.g., Shalley et al., 2000). Secondly, researchers can collect the data on the predictors of employee innovative behavior and employee innovative behavior itself at two different points of time (Podsakoff et al., 2003). For instance, the data on inclusive leadership were collected at T1, whereas the data on employee innovative behavior were collected at T2. Thirdly, developing a new measurement of creative self-efficacy should be encouraged in future research. Last, studies also could use longitudinal designs or quasi-experimental to further improve the accuracy of conclusions.

Secondly, this study is conducted in China which is considered to have a characteristic of high-power distance culture. Under high-power distance culture, the extent to which inequality among people in different positions of formal power is viewed as a natural aspect of the social order (Brockner et al., 2001). Zhang et al. (2016) argued that high-power distance culture weakens the effectiveness of inclusive leadership by hindering the development of the benign relationship between subordinates and their superiors. Thus, in future research, culture can be considered as a moderating variable to reveal the boundary conditions of the influence of inclusive leadership on employee innovative behavior. Furthermore, both the positive effect mechanism and negative effect mechanism of inclusive leadership must be studied.

Finally, the present study only examines the positive effect of inclusive leadership; however, the relationship between inclusive leadership and employee innovative behavior may be non-linear. Future researches can explore the curvilinear relationship between inclusive leadership and employee innovative behavior.

## Data Availability Statement

The original contributions presented in the study are included in the article/Supplementary Material, further inquiries can be directed to the corresponding author/s.

## Ethics Statement

The protocol was approved by an institutional review board of Xiangtan University of China. All subjects read informed consent before participating this study and voluntarily made their decision to complete surveys.

## Author Contributions

HW developed the theoretical model, collected the data, and wrote the manuscript. MC and XL collected the data and analyzed the data. All authors contributed to the article and approved the submitted version.

## Conflict of Interest

The authors declare that the research was conducted in the absence of any commercial or financial relationships that could be construed as a potential conflict of interest.

## Publisher's Note

All claims expressed in this article are solely those of the authors and do not necessarily represent those of their affiliated organizations, or those of the publisher, the editors and the reviewers. Any product that may be evaluated in this article, or claim that may be made by its manufacturer, is not guaranteed or endorsed by the publisher.
